# Viruses and Bacteria Associated with Cancer: An Overview

**DOI:** 10.3390/v13061039

**Published:** 2021-05-31

**Authors:** Davide Zella, Robert C. Gallo

**Affiliations:** 1 Institute of Human Virology and Global Virus Network Center, Department of Biochemistry and Molecular Biology, University of Maryland School of Medicine, Baltimore, MD 21201, USA; dzella@ihv.umaryland.edu; 2 Institute of Human Virology and Global Virus Network Center, Department of Medicine, University of Maryland School of Medicine, Baltimore, MD 21201, USA

**Keywords:** bacteria, viruses, carcinogenesis, cancer progression, anti-cancer therapy, DnaK

## Abstract

There are several human viruses and bacteria currently known to be associated with cancer. A common theme indicates that these microorganisms have evolved mechanisms to hamper the pathways dedicated to maintaining the integrity of genetic information, preventing apoptosis of the damaged cells and causing unwanted cellular proliferation. This eventually reduces the ability of their hosts to repair the damage(s) and eventually results in cellular transformation, cancer progression and reduced response to therapy. Our data suggest that mycoplasmas, and perhaps certain other bacteria with closely related DnaKs, may also contribute to cellular transformation and hamper certain drugs that rely on functional p53 for their anti-cancer activity. Understanding the precise molecular mechanisms is important for cancer prevention and for the development of both new anti-cancer drugs and for improving the efficacy of existing therapies.

## 1. Introduction

The six biological capabilities comprising the hallmarks of cancer, namely sustaining proliferative signaling, evading growth suppressors, resisting cell death, enabling replicative immortality, inducing angiogenesis, and activating invasion and metastasis constitute a logical framework for understanding the diversity of neoplastic diseases. Either by themselves or in combination with other co-factors, certain viruses and bacteria are able to cause cancers by affecting these important cellular pathways. A comprehensive review of them is outside the scope of this report. We review here the most known viruses and bacteria currently associated with cellular transformation and provide a summary of their proposed mechanisms of action.

## 2. Mechanisms of Cellular Transformation

The employment of new technologies in molecular analysis, namely gene expression profiling, protein networks, microRNA expression and pathway analysis and gene discovery, indicates that carcinogenesis is derived mostly from the clonal proliferation of a single initiating cell. However, recent evidence shows evidences of a multiclonal origin. The proliferating cells eventually become phenotypically different from the surrounding normal parenchyma.

Several experimental data both in vitro and in vivo, have demonstrated multistage models of carcinogenic processes in several organ systems, such as skin, liver, urinary bladder, lung, kidney, intestine, mammary gland, and pancreas. These models allow us to classify various agents as initiators, promoters, and complete carcinogens and are to define phases of carcinogenesis—initiation, promotion, and progression. However, it is worth noting that each one of these different phases may indeed be subdivided into multiple stages. Accordingly, the current multistage model of carcinogenesis involves a morphological continuum spanning from hyperplasia/preneoplasia to carcinoma, and it mainly includes the alterations of four broad categories of cancer-related genes, namely defective DNA repair genes, the activation of oncogenes, the inactivation of tumor suppressors that affect cell proliferation, and the reduction/inhibition of genes involved in apoptosis. During this multistep process, each neoplasm, deriving from the original single cell, accrues numerous mutations totaling at least 80 genetic alterations in cancer-related genes, a dozen of which are generally considered “driver” mutations [1]. In addition, extensive cytogenetic alterations are also frequently observed in cancer cells [2,3]. As a result of this genomic diversity, important cellular modifications are observed, including limitless replicative potential, sustained angiogenesis, and the ability to invade and metastasize.

It is thus not surprising that a number of studies indicate that essentially all of the viral proteins of oncogenic viruses exert their carcinogenic activity by altering the functions of one or more of these important cellular pathways. The mechanistic studies regarding the association of bacteria and cancer are so far less abundant. Two important reasons for this are that: (i) the potential effectors are vastly more abundant and consequently more difficult to determine, and (ii) there are limited animal models available. However, most recent data including our own indicate that some bacteria associated with cancer exert their transforming activity by expressing proteins that are indeed affecting the same cellular mechanisms targeted by oncogenic viruses. Therefore, what was seen as a patchwork of unrelated effectors may now be grouped together by a common basic theme, namely their ability to interfere with proteins involved in DNA repair, proliferation and/or apoptosis of the cellular host. We review here the most known oncogenic viruses and bacteria associated with cancer according to this unifying theme.

## 3. Viruses Associated with Cellular Transformation

There is a well-known association of certain DNA and RNA viruses with cancers, namely HTLV-1, HPV, HBV, HCV, EBV and HHV-8. We note that HTLV-1 is the most oncogenic of the list, followed by HPV, HBV and HCV. EBV and HHV-8 for the most part require a co-factor to exert their oncogenic potential, but as global health problems they have a very large impact because of their far greater prevalence. Their viral genomes have a reduced size (from a few Kb to about 200 Kb) and consequently a reduced coding ability. Thus, they depend on cellular proteins to complete their life cycles and increase the production of viral particles and in doing so they affect several cellular pathways including DNA repair, proliferation and apoptosis [4]. As a consequence, these viruses promote oncogenesis by a multi-steps process, which involves tumor initiation and/or later stage tumor promotion and spreading, like (but not limited to) regulation of cell proliferation, apoptosis and senescence. Indeed, different viruses can affect different stages of tumor formation, even though the process is not virus-specific. For the most well-known oncogenic viruses we will briefly summarize the major identified mechanisms, which promote different stages of tumor development.

### 3.1. HTLV-1

The first known human retrovirus, human T-cell leukemia virus type1 (HTLV-1) is an RNA tumor virus [5] and it has been the one most convincingly linked to a cancer, namely adult T-cell leukemia/lymphoma [ATL]. A peculiar property of HTLV-1, which makes it stand out among other microbiological agents, is its ability to cause transformation without requiring any know cofactor [5,6,7,8,9].

The major transmission routes are through sexual contact and parenterally (i.e., blood transfusions, needles and through breastfeeding).

The World Health Organization classifies HTLV-1 as one of the most potent oncogenic agents, with about 5% of cases developing ATL leukemia after a very long incubation time post-infection [10]. An estimated 5 to 10 million people are infected worldwide, it is endemic in some Africa countries and it is mainly detected in certain areas of the Caribbean (probably due to a founder effect from descendants of African tribes), in areas of southern Japan, in natives of Australia and in Iran [11]. In the rest of the world its presence is identified (for example Peru, Brazil and Romania) but not clearly calculated for the lack of precise data (https://www.ecdc.europa.eu/sites/portal/files/media/en/publications/Publications/geographical-distribution-areas-high-prevalence-HTLV1.pdf. Stockholm, March 2015).

HTLV-1 is also the etiological agent of the HTLV-1-associated myelopathy/tropical spastic paraparesis (HAM/TSP), that is believed to originate from an abnormal immunological response following host–virus interactions, mainly observed in the Caribbean region [12,13].

HTLV-1 has a genome of about 9kb nucleotides, and it is structured with the classical retroviral genes, i.e., gag, pol, and env, with an additional region called pX. This region encodes for a number of regulatory proteins (including Tax and Rex). Both these proteins are necessary for viral replication, and in addition Tax also functions as a transcriptional trans-activator, which controls viral and cellular gene expression and it enhances cell proliferation. Several data indicate that Tax is arguably the most important protein of HTLV-1 involved in cellular transformation, although to date the precise molecular mechanism(s) responsible for genomic abnormalities in HTLV-I-infected cells is still largely unknown [14,15,16]. HTLV-1 Basic leucine-Zipper factor (HBZ) is another protein with oncogenic properties able to induce T cell lymphoma in vivo [17,18]. HBZ is constitutively expressed in ATL patients and it is regulated by the 3’ LTR [19], and it has been demonstrated to interfere with a number of cellular functions, including proliferation and apoptosis [20]. It should be noted that although Tax seems to play an essential role in early steps of oncogenesis, it may not be essential for maintenance (as HBZ). Indeed many tumors are tax negative. Nonetheless, it appears that both Tax and HBZ are likely synergizing in their cancer-promoting ability, and this may explain the highly oncogenic potential of HTLV-1.

### 3.2. HPV

HPVs belong to the Papillomaviridae family and are non-enveloped dsDNA viruses with an 8 kb long genome. Human papillomaviruses (HPVs) are associated with several human cancers, and the observations date back in the 1970s [21,22,23]. The route of transmission is primarily through skin-to-skin, skin-to-mucosa and mucosa-to-mucosa contact. 

In the United States, HPV causes about 3% of overall cancers in women and about 2% of overall cancers in men, although this percentage is increased for some cancers, namely cancers of the oropharynx, vulva, vagina, penis, anus and anogenital tract (>50% of cancers in females, about 5% in males) [24]. Upon initial infection of undifferentiated cells in the basal layer of stratified epithelium, the HPV genome remains in an episomal form and the different viral stages of genome amplification, expression of its late genes and ultimately whole virus assembly is performed in differentiated suprabasal epithelial cells.

Characterization of viral subtypes allowed identification and association of HPV16 and HPV18 with cervical carcinoma [25], followed by the demonstration of their causal role [24]. The oncogenic properties of “high-risk” HPVs (so far a total of 12 HPVs are classified as high risk including HPV16, −18, and −31, −33, 35. 39, 45, 51, 52, 56, 56, 59) are believed to be linked mostly to the proteins E6 and E7 [26,27,28,29,30], while in most cases viral insertion leads to lack of the regulatory protein E2.

HPV is believed to cause cancer by several mechanisms. One of them is integration of its DNA into the host genome, thus altering important cellular functions, which eventually promote transformation. A second mechanism regards some of the early genes expressed by HPV acting as oncogenes [31].

### 3.3. HBV and HCV

Hepatitis B Virus (HBV) and Hepatitis C Virus (HCV), the two Hepatitis viruses, are different both in genomic composition and structure. HBV, a member of the Hepadnaviridae family, has a partial double strand DNA genome about 3.2 kb nucleotides long. On the other hand, the genome of HCV is a single-strand RNA of about 9.6 kb nucleotides long, translated inside the cells into a single protein and then further processed into 10 proteins. HBV major routes of transmission are through the bodily fluids of an infected person, including blood-to-blood contact, sweat, saliva, tears, vaginal secretions, menstrual blood, breast milk and semen. HCV is mainly transmitted through blood transfusion and injection drug use, while the likelihood of sexual transmission is very low. However, their high prevalence points to other still undiscovered route(s) of transmission. Both HBV and HCV are responsible for chronic liver infections, cirrhosis, liver failure and hepatocellular carcinoma (HCC) [32,33]. Though the precise molecular mechanisms of how hepatitis viruses cause cancer are largely unknown, there are several driving forces contributing to hepatocytes transformation caused by the two viruses, including chronic inflammation, DNA damage and epigenetic modifications [34,35]. In addition, several viral proteins have been implicated in directly modulating cellular pathways that promote malignant transformation [36], and among them we note: (i) HBV-expressed HBX, which binds to p53 and blocks its anticancer functions and also prevents its association with several transcription factors of the DDRs; and (ii) Nonstructural Protein 5A (NSP5A), which has been correlated to HCV-mediated reduction of DNA-repair-related mechanisms.

### 3.4. EBV (HHV-4) and HHV-8 (KSHV)

Epstein–Barr Virus (EBV), or human gammaherpesvirus 4 (HHV-4), is a DNA virus, and it was first observed by electron microscopy in Burkitt’s lymphoma cells [37]. EBV spreads most commonly though bodily fluids, especially saliva. Other minor but significant routes of transmission are through blood and semen, and using objects used recently by an infected person. All this makes EBV extremely widespread, and in fact more than 90% of people are infected by EBV in their youth, showing the almost ubiquitous diffusion of EBV infections [38]. EBV is a very poor and inefficient carcinogenic agent [39,40]; nonetheless, its ubiquity makes it important as it contributes to about 1.5% cases of human cancer worldwide. Proteins belonging to the families of Epstein–Barr nuclear antigens (EBNAs) and Latent Membrane Proteins (LMPs) have been mainly implicated in the oncogenic properties of EBV [41].

Another DNA virus belonging to the gamma herpes virus family is Human herpesvirus 8 (HHV-8) or Kaposi’s sarcoma-associated herpesvirus (KSHV). Its exact diffusion is not known, but it is thought to be widespread, particularly in Africa. It is believed to spread mainly through saliva, thought also sexual contact has been proposed. Though involved in cellular transformation, HHV-8 has comparably poor transforming capabilities [42]. In fact, it is rarely associated with cancer, except in HIV-positive subjects where it represents a significant contribution to the number of cancer cases, suggesting the involvement of HIV as a cofactor for transformation [43].

Both viruses share a latent phase when very few proteins are expressed and a lytic one, when they are actively replicating. Depending on both the site of infection and the viral phase, several types of disease are observed. For example, when both viruses establish latent infection in the B-cells, they are associated to lymphomas. Conversely, while no cancers are associated with reactivation of EBV, HHV-8 reactivation in lymphatic endothelium is associated with Kaposi Sarcoma.

Finally, we note the presence of genes codifying for chemokines agonists or antagonists of chemokine activity, or chemokine-receptor homologues. Both components of viral mimicry, which are expressed by infected cells, might reduce the chemokine concentration near the infected cell, transmit signals inside the cell that promote viral replication, or confer on the infected cell the ability to migrate to other tissues, thus potentially contributing to oncogenesis.

### 3.5. Merkel Cell Polyomavirus (MCV)

Merkel Cell Polyomavirus (MCV) was identified in 2008 (Feng et al., 2008) in a rare but aggressive neuroendocrine tumor (Merkel cell carcinoma, MCC) of the skin. In the US, there are about 1600 new cases annually of MCC, the 5-year mortality rate is about 46% and the annual incidence is about 0.6 per 100,000 persons (0.06%). The median age at diagnosis is >70 years, while it is rarely detected below 50 year (about 4% of cases) and is extremely rare in children. The oncogenic mechanism of MCV is not yet clear, although MCC increase upon UV irradiation indicates a likely involvement of defective DNA repair mechanism(s) in the transformation process. Moreover, the increased incidence of MCC observed in AIDS individuals or solid organ transplant recipients, together with reports showing tumor regression upon increased immune functions, indicates that a weakened immune system may be related to the onset of MCC. In summary, it seems that MCV needs other co-factors and loss of immune-surveillance to induce MCC, and further data are needed to evaluate its oncogenic potential.

## 4. Bacteria Associated with Cellular Transformation

*Helicobacter pylori* so far is the only bacterium with clear epidemiological data that support a causal link with carcinogenesis [44], though an increasing number of bacteria have been associated with human cancers and studies of the human microbiome have elucidated an array of complex interactions between prokaryotes and their hosts [45]. Although the detailed molecular mechanisms employed by these bacteria to influence the cellular pathways leading to transformation are still largely unknown, accumulating evidence indicate that they have the potential to both hamper p53 activities and affect pathways of DNA repair, increasing accumulation of DNA damage, and eventually driving cellular transformation antitumor progression [46,47,48].

Additional evidence of bacterial involvement in carcinogenesis was provided by studies in animal models showing a reduction in tumor load after antibiotic manipulation of gut microbiota [49]. Consistent with this idea, in vivo experiments in several chemically induced or genetically deficient mouse models showed that germ-free conditions reduce colonic tumor formation [50,51]. In more detail, several studies in patients have highlighted an associations between *Fusobacterium nucleatum* and colorectal cancer [52,53,54,55,56,57,58], *Chlamydia trachomatis* and cervical cancer [59,60,61,62,63,64], mycoplasmas and prostate and colorectal cancer, as well as non-Hodgkin’s lymphoma (NHL) in HIV-seropositive subjects [65,66,67,68,69,70]. In addition, infections with several mycoplasmas (*Mycoplasma fermentans*, *arginini*, *hominis* and *arthritidis*) inhibit p53 activity and cooperate with Ras, leading to oncogenic transformation in vitro, although the responsible bacterial protein has not been yet identified [71], strongly supporting them as leading bacterial candidates with oncogenic properties. It has also been shown that persistent infection with *M. penetrans* in a chemically immunosuppressed mouse model resulted in reduced p53 and p21 expression in gastric mucosal cells, leading to pathological changes [72]. These findings indicate that, in some cases, mycoplasmas could facilitate tumorigenesis, though (as mentioned above) no direct carcinogenic roles for any mycoplasmas have been demonstrated in vivo. Additionally, *M. fermentans* subtype *incognitus* induces chromosomal alterations in vitro that result in phenotypic changes, leading to acquisition of malignant properties in mouse and human cells, including loss of anchorage dependency, ability to form colonies in soft agar, and tumorigenicity in nude mice [68,69,70,73]. Recent reports highlight the presence of tumor-specific bacteria comprising the so-called tumor microbiome, and the influence of these bacteria on tumor progression and anti-cancer therapy is being actively investigated [74,75,76]. To avoid host cell clearance bacteria use a number of strategies to accomplish both immune-evasion and immune cell elimination, resulting in replication and spreading. Moreover, a number of bacteria, in particular the ones most notably associated with cellular transformation, developed molecular mechanisms to achieve persistent intracellular infection.

Our recent data showed that a protein present in a subspecies of *M. fermentans*, DnaK, belonging to the HSP70 chaperon family, reduced PARP1 activity triggered by DNA damage [77,78]. PARP1 belongs to the PARP protein family [79], and upon recognition of single and double-strand DNA breaks, acts by modifying (PARylating) and in turn activating certain proteins including, among others, PARP1 itself, DNA-dependent protein kinase (DNA-PK), topoisomerase 1 (TOP1), and histones, recruiting them to the site of damage to ensure proper repair [80,81]. Inaccurate DNA repair typically results in apoptosis, to prevent accumulation of DNA damage, eventually leading to cellular transformation. Our data also show that Mycoplasma DnaK co-immunoprecipitates with USP10, a key p53 regulator, and this resulted in a reduction of p53-dependent anti-cancer functions, hampering the effect of drugs that rely on p53 activation to exert their anti-cancer effect [78]. Moreover, phylogenetic analysis demonstrated high similarity in amino acid composition among DnaKs from certain bacteria associated with human cancers (including certain Mycoplasmas, *H. pylori, F. nucleatum* and *C. thrachomatis*), suggesting a likely common mechanism of cellular transformation [78]. Finally, we demonstrated that exogenous DnaK induces improper protein phosphorylation [82], adding to the current knowledge information on the role of bacteria in the tumor microenvironment in deregulating cell functions to ultimately promote cancer progression [83]. Taken together, our data thus indicate that certain bacteria with closely related DnaK are potentially involved in cellular transformation, tumor progression and resistance to cancer therapy by altering DNA repair mechanisms and anti-cancer drug activity.

## 5. Conclusions

This review aims to summarize some of our current knowledge of cellular transformation and cancer progression induced by viruses and bacteria and attempts to join previously described virus-related mechanisms with newly discovered bacterial-related mechanisms. While a number of viral proteins involved in carcinogenesis have been identified, the detailed molecular mechanisms employed by some bacteria to influence the cellular pathways leading to transformation are still largely unknown. However, a number of studies indicate that a common picture is emerging, namely that some cancer-associated bacteria are similar to oncogenic viruses in their transforming ability, because they also express proteins that are able to affect a number of important cellular proteins, eventually altering DNA repairs, cell cycle/proliferation and apoptosis. This in turn increases the accumulation of DNA-damage, which eventually results in cellular transformation, tumor progression and reduced response to therapy. By harnessing the knowledge acquired with the study of oncogenic viruses, it is thus likely that the precise identification and characterization of the molecular mechanism(s) of action employed by cancer-associated bacteria will contribute to further clarify the similarities with oncogenic viruses. This will not only improve our knowledge of the origin of cancer, but it will also have preventative, diagnostic and therapeutic implications.
